# Supplementary material for: Positive Effects of Land Use Change on Wintering Bar-Headed Geese between 2010 and 2021
Source: Animals (Basel). 2022 Nov 14;12(22):3142. doi: 10.3390/ani12223142 (PMC9686978; doi:10.3390/ani12223142)
Supplement: Supplementary file 1 [file animals-12-03142-s001.zip › animals-1990051-supplementary.pdf]

Table S1. the six variables including the croplands, grasslands, open water, temperature, precipitation and wind velocity.

Year	Temperature (°C)	Precipitation (mm)	Wind velocity (m/s)	Cropland (km^2)	Open water (km^2)	Grassland (km^2)
2010	5.2	12.85	2.4	211.67	22.29	22.58
2012	6.2	2.65	2.65	210.37	22.21	23.5
2013	4.5	11.05	2.1	204	22.1	27.31
2014	4.4	16.8	2.35	201.46	22.19	27.82
2015	7	8.05	2.2	196.94	22.4	30.47
2016	6.9	14.05	2	193.66	22.63	32.11
2017	5.35	5	1.7	190.8	22.88	33.51
2018	6.45	5.55	2.05	188.43	23.3	35.04
2019	6.35	6.95	1.95	187.82	23.35	35.4
2020	5	4.4	1.8	188.56	23.54	34.45
2021	5	13.15	1.95	189	23.52	33.52
Min	4.4	2.65	1.7	187.82	22.1	22.58
Max	7	16.08	2.65	211.67	23.54	35.4
Mean	5.7	9.14	2.10	196.61	22.76	30.52

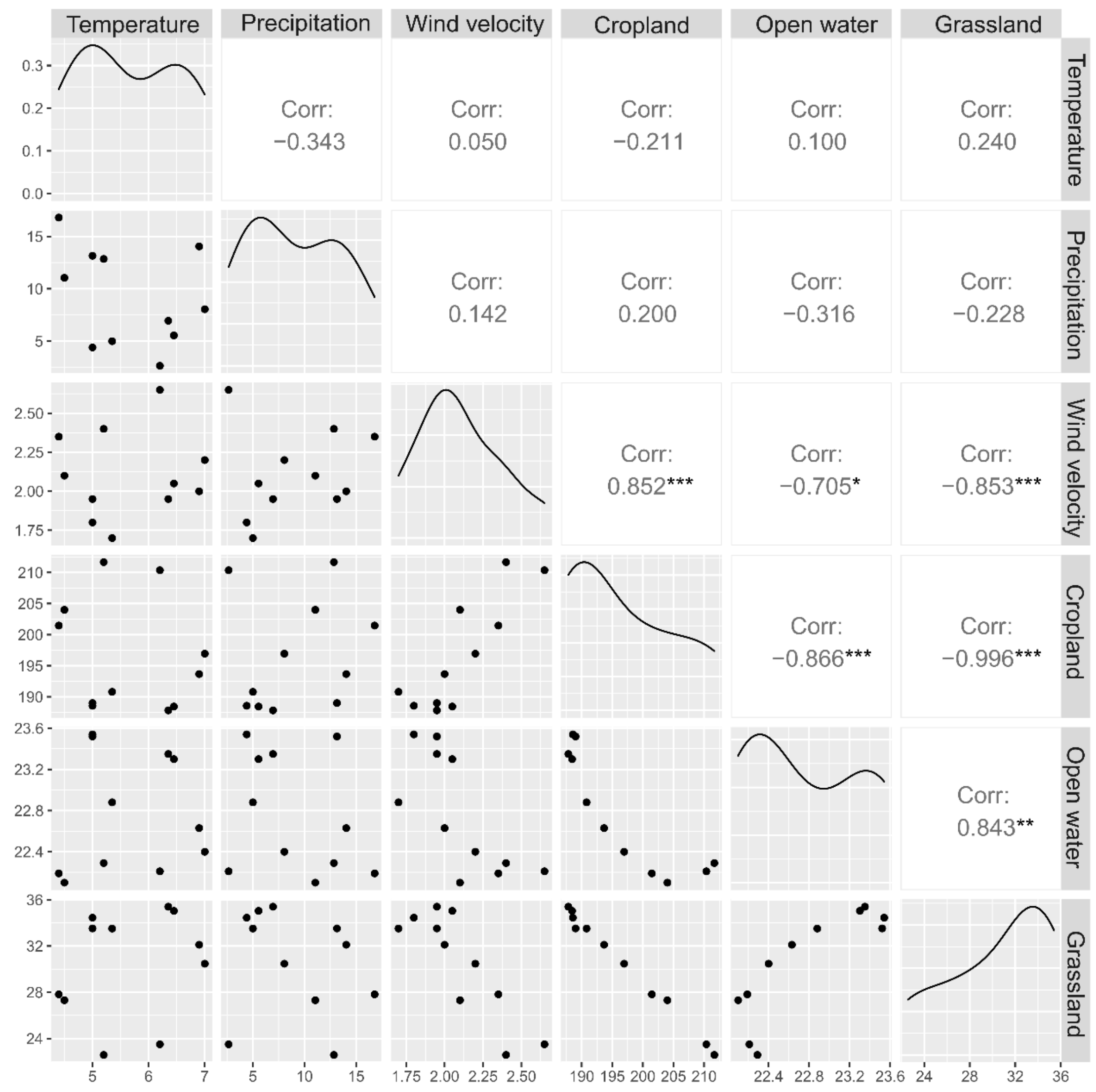

Figure S1. Correlation analysis Pearson's correlation coefficients among the variables of Cropland, Grassland, Precipitation, Temperature, Open water and Wind velocity.

Table S2. variance inflation factor (VIF) of the six factors.

	Temperature	Precipitation	Wind velocity	Cropland	Open water	Grassland
VIF	1.77	1.86	5.15	311.3	7.71	270.55
VIF<10	TRUE	TRUE	TRUE	FALSE	TRUE	FALSE

Table S3. Results of Model selection based on linear models. The top 10 models ranked by criterion corrected for small sample sizes (AICc). Predictive variables include Precipitation, Temperature, Open water, Wind velocity. NA indicates that the predictive variable is not included in the given model.

Precipitation	Temperature	Open water	Wind velocity	R^2	logLik	AICc	ΔAICc	weight
NA	NA	0.839	NA	0.7	-0.750	10.929	0.000	0.795
NA	NA	0.811	-0.041	0.7	-0.735	16.136	5.207	0.059
NA	0.025	0.836	NA	0.71	-0.739	16.145	5.216	0.059
-0.006	NA	0.838	NA	0.7	-0.749	16.165	5.237	0.058
NA	NA	NA	-0.614	0.37	-4.862	19.153	8.224	0.013
NA	NA	NA	NA	0	-7.459	20.419	9.490	0.007
NA	0.032	0.800	-0.049	0.7	-0.717	23.434	12.506	0.002
-0.012	NA	0.806	-0.043	0.71	-0.732	23.465	12.536	0.002
0.001	0.025	0.836	NA	0.71	-0.739	23.478	12.549	0.001
-0.167	NA	NA	-0.605	0.4	-4.611	23.888	12.960	0.001
